# Effects of different doses of synthetic oxytocin on neonatal instinctive behaviors and breastfeeding

**DOI:** 10.1038/s41598-022-20770-y

**Published:** 2022-09-30

**Authors:** Yun Zhou, Wenwen Liu, Yang Xu, Xiaoyan Zhang, Yiqun Miao, Aihua Wang, Yuanyuan Zhang

**Affiliations:** 1 School of Nursing, Weifang Medical University, Weifang, 261053 Shandong Province China; 2 School of Nursing, Qilu Medical University, Zibo, 255300 Shandong Province China; 3 Delivery room, Affiliated Hospital of Weifang Medical University, Weifang, 261035 Shandong Province China

**Keywords:** Health care, Medical research

## Abstract

Synthetic oxytocin is the current domestic first-line agent of induced labor and labor augmentation, and its potential effects on neonatal neurobehavioral development is currently attracting increased attention. To explore the effect of different doses of synthetic oxytocin on neonatal instinctive breastfeeding behavior and breastfeeding by observing neonatal behaviors during skin-to-skin contact with mothers after delivery. Observations and comparisons of neonatal instinctive behaviors were conducted by using Widström's 9 Stages method. According to the total dosage of oxytocin administered during labor, participants were divided into a low dose group (≤ 2.5 U) of 39 pairs, a medium dose group (> 2.5 U) of 38 pairs, a high dose group (> 7.5 U) of 38 pairs and a control group (no synthetic oxytocin use) of 39 pairs. The occurrence time of newborns' instinctive movements and the duration of each behavior stage for the four groups were also analyzed. The number of exclusive breastfeeding sessions within 3 days after birth and the rate of exclusive breastfeeding at 3 months were collected and compared. There were significant differences among the four groups in the occurrence time of raising head or turning head (*p* = 0.004), eating hands (*p* = 0.011), moving body (*p* = 0.001), locating areola (*p* < 0.001), licking nipples (*p* = 0.002), containing nipple (*p* = 0.001), sucking (*p* < 0.001). There were significant differences among the four groups in the duration of activity (*p* = 0.004), clawing (*p* = 0.001), familiarization (*p* = 0.001), and sucking (*p* < 0.001). There was also a significant difference in the number of exclusive breastfeeding sessions of 24 h (*p* = 0.011), 48 h (*p* < 0.001), 72 h (*p* = 0.001) after birth among the four groups, but there was no statistical difference in the rate of exclusive breastfeeding at 3 months after birth. The intrapartum administration of synthetic oxytocin was associated with the expression of neonatal instinctive breastfeeding. With increases in drug dose, the effect of breast seeking activity and breast attachment was more significant, and the association of synthetic oxytocin on sucking and breastfeeding was dose-dependent.

## Introduction

In the 1950s, Du Vigneaud et al.^1^ developed a synthetic version of oxytocin that could control hormones during administration. However, synthetic oxytocin (SynOT) is one of 12 drugs that are designated as a "high-alert medication" by the Institute for Safe Medication Practices. SynOT has received a "black box" warning from the United States Food and Drug Administration (FDA), which indicates that it should not be used for labor initiation without medical indications^2,3^. SynOT can promote cervical softening, maturation and uterine contractions in its role as the current first-line agent of induced labor and labor augmentation. Factors such as cervical maturity and individual sensitivity to the drug may lead to differences in the required concentrations and doses^4^. However, high doses of synOT may increase the potential effects on mothers and newborns.

An increasing number of studies have focused on the adverse effects of synOT on newborns and mothers. For example, increased stimulation of the uterine contractions with synOT may cause an even more excessive uterine tissue production of lactate. The level of lactate in amniotic fluid rose in line with the increasing dosage of synOT, indicating a more anaerobic environment in the contracting uterine muscle, leading to a decrease in umbilical artery PH and an increased probability of neonatal acidemia and neonatal hypoxia^5,6^. Exposure to synOT during labor may affect the maternal endogenous oxytocin system, and may affect the maternal stress reactivity, mood and mothering behaviors, including lactation and breastfeeding^7–12^. Correlational findings have suggested that perinatal synOT may affect the developing behavioral systems of young children^13^, and an inverse correlation has been found between synOT exposure and the level of prefeeding organization at one hour after birth^14^. SynOT may inhibit the expression of primitive reflexes favoring breastfeeding initiation, especially rhythmic reflexes, such as sucking, jaw jerking and swallowing^15^. Further research is needed to support whether oxytocin could affect other neonatal behaviors.

Widström et al. observed that healthy full-term newborns exhibit instinctive behaviors when they experience skin-to-skin contact (SSC) with their mothers immediately after labor. If undisturbed, infants can move to the mother's breast and start sucking within 2 h after birth^16,17^. Widström divided these instinctive behaviors into Birth cry, Relaxation, Awakening, Activity, Rest, Clawing, Familiarization, Suckling, Sleeping stage^17^ (Table 1), which provides conditions for observing instinctive breastfeeding behavior of newborns after birth. The full expression of instinctive behaviors can not only promote the success of early breastfeeding, but also contribute to the good interaction and the establishment of neonatal security. It also reflects early neonatal neurodevelopment and has an important influence on neonatal intelligence development and psychosocial development^18^. Therefore, this study explored the impact of different doses of synOT on neonatal instinctive breastfeeding behavior.

**Table 1:** Widström's 9 stages.

Birth cry	Infant has intense and specific cry just after birth
Relaxation	Newborn has a rest and recovery time immediately after the cry ends, and newborn has no activity
Awakening	At awakening, newborn begins to show signs of activity. Newborn opens their eyes and has limited movements of mouth, hands and shoulders
Activity	Apparent movement of the head, arms and shoulders of the newborn; newborn has rooting activity
Rest	Rest could occur during each stage or in between; newborn has some mouth activity
Clawing	Newborn moves toward breast and nipples by pushing, bobbing and shifting body
Familiarization	Newborn has moved around the areola area, licked and tasted the nipples and areola
Suckling	Newborn has contained the nipple by self and has begun breast-feeding
Sleeping	Newborn has closed eyes approximately 1.5–2 h after birth

## Methods

### Participants

Our observational study was conducted in the Affiliated Hospital of Weifang Medical College and has been approved by the Ethics Committee of Weifang Medical College. We recruited women who came into the delivery room for labor from July 2020 to October 2020. All participants were performed following relevant hospital guidelines and regulations, without interventions other than routine treatment measures in the delivery room. Women who met the following criteria were included : (1) those who were > 20 years of age and vaginal delivery; (2) single pregnancy and 37–42 weeks pregnant. This included women those who chosed to induce labor due to personal and family wishes or near postterm pregnancy, and those who had spontaneous labor without synthetic oxytocin; (3) personal and family support and planning for breastfeeding. However, (1) women selected labor analgesia; (2) used drugs other than oxytocin during labor; (3) with pregnancy complications or complications; (4) vacuum-assisted vaginal birth or forceps were used during labor; (5) postpartum bleeding(> 500 ml within 24 h after delivery); (6) infants’ Apgar score < 8 in 1 min after birth; (7) infants’ weight < 2500 g or ≥ 4000 g; (8) infants suffering from genetic diseases or malformations; (9) mothers refuse SSC or separation of mother and baby occurs during SSC were excluded. Informed consent was obtained from all participants who met the inclusion and exclusion criteria before observation. Incomplete data or lost respondents were considered drop-outs and lost to follow-ups.

### Procedure and measures

All of the healthy newborns (delivered vaginally) were encouraged to have SSC with their mother’s chest for at least 90 min immediately after labor. After labor, the newborn was quickly wiped dry and placed naked on the mother's chest. The newborn was then covered with a blanket, as well as wearing a hat to keep warm. Before the study implementation, two researchers were educated through professional videos and workshops provided by the research group on the 9 stages of Widström's neonatal behavior to ensure that the researchers were able to identify instinctual behavioral movements in each stage^17^. While ensuring the safety of the mother and baby, researchers carefully observed neonatal instinctive behaviors, taking the average of the recording time of the two researchers. Observation continued until the newborn closed eyes and fell asleep. If the newborn failed to breastfeed successfully, the observation ended 90 min after skin-to-skin contact. During the entire skin-to-skin contact process, no intervention was added to the newborn's movements.. After observation, the newborns were given routine procedures, such as vitamin K1 injections and weighing. The researchers examined the medical records of the newborns and mothers and collected demographic data, as well as the dosages of synthetic oxytocin. The mothers and infants were grouped according to the dose of synthetic oxytocin that was administered.

The administration of SynOT in this study was in accordance with the unified standard of Chinese guidelines^19^: the intravenous infusion of SynOT should be gradually increased starting at a low dose. Using an infusion pump, the initial dose of 2.5U synthetic oxytocin in 500 ml of normal saline was begun at 8 drops per minute (2.7 mU/min) and increased by 8 drops every 20 min. Before making adjustments, medical professionals had to observe the contractions for at least 10 min. Additionally, the maximum drop rate should not exceed 40 drops per minute (13.2 mU/min). If there were still no effective uterine contractions (3 contractions occurring within 10 min, each lasting 30–60 s) when the maximum drop rate was reached, dissolved 5U synthetic oxytocin in new 500 ml normal saline when the liquid in the bag was about to be finished, and the new bag would be replaced after the last bag was finished. The dropping rate was first reduced by half after replaced to 20 drops per minute (13.2 mU/min), then adjusted according to the intensity of uterine contractions, and the maximum drop rate were increased to 40 drops per minute (26.4 mU/min). 5U of synthetic oxytocin was still dissolved in fresh 500 ml of normal saline if it were essential to continue the drug, and the infusion was continued until effective uterine contractions took place. The use of synthetic oxytocin was gleaned from the medical records after observation: 2.5 U was utilized as the initial dose, followed by a low-dose group of ≤ 2.5 U, a medium-dose group of > 2.5 U, a high-dose group of > 7.5 U, and a control group of no SynOT. .

The number of exclusive breastfeeding sessions at 24 h, 48 h, 72 h postpartum in each group were collected. During the outpatient review 3 months after delivery, the mothers were asked whether the mode of breast-feeding was exclusive breast-feeding, mixed feeding or artificial feeding, calculate the pure breastfeeding rate.

### Statistical analysis

IBM SPSS Statistics 26.0 (https://www.ibm.com/analytics/spss-statistics-software) was used for the statistical analysis. Measurement data were statistically described via means ± standard deviations. An analysis of variance (ANOVA) was used to compare the occurrence of neonatal instinctive movements and duration of behavior stage in each group, and the number of exclusive breastfeeding sessions. Further pairwise comparisons were performed by using the Bonferroni correction. Frequencies and percentages were used for the statistical description of the classification data, and the chi-square test was used for the analysis. *P* < 0.05 was considered to be statistically significant.

## Results

**Figure 1:**
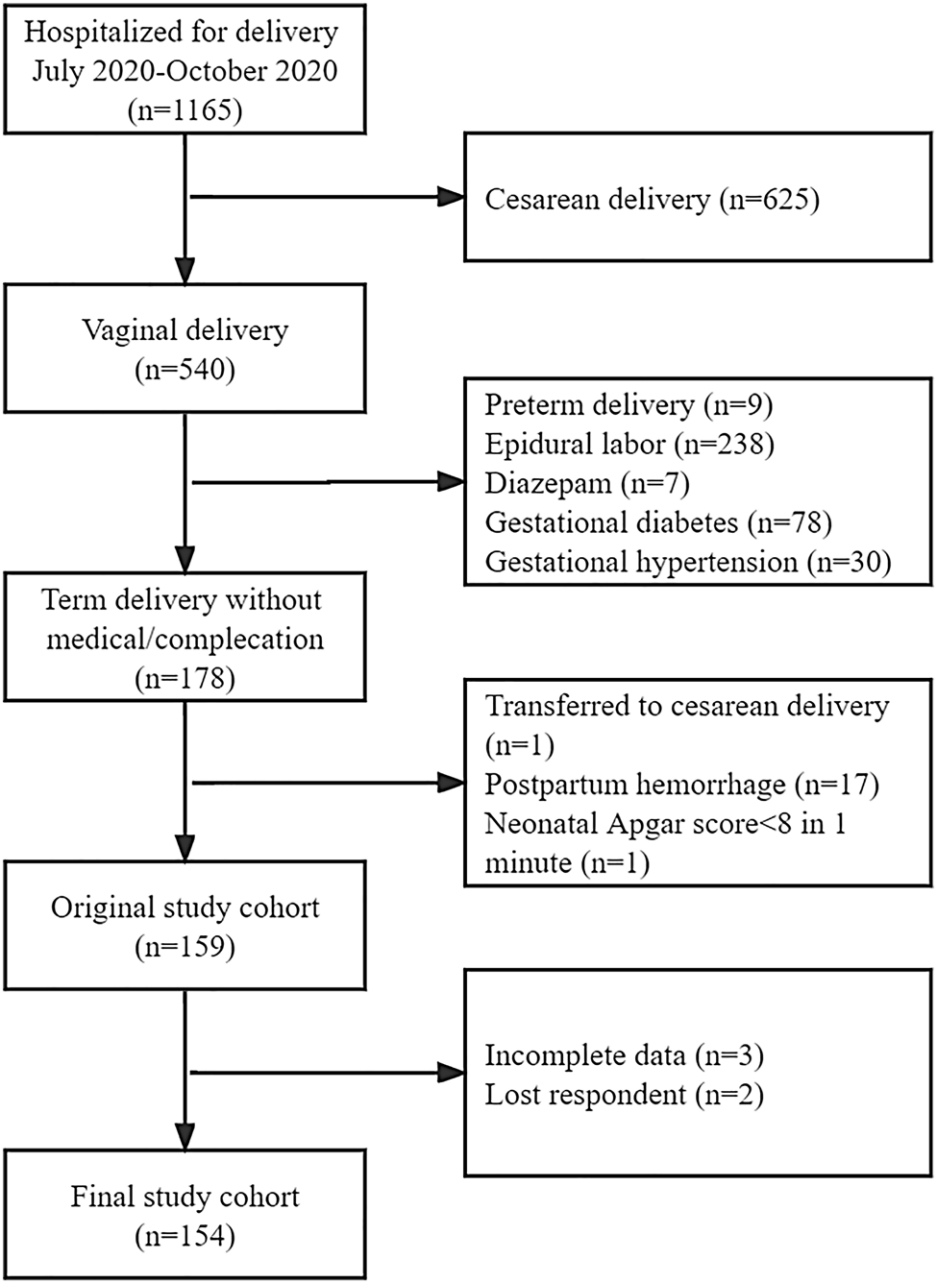
Recruiting fow diagram. *Notes*: *P < 0.05, **P < 0.01, ***P < 0.001; if the letters marked at the top of the bar chart were different, there was a statistically significant difference between the two groups. Otherwise, there was no difference.

During the study period, 1,165 women were admitted to our hospital for delivery, and 540(46.4%) opted for vaginal delivery. Among these, 362(67.0%) did not meet the study inclusion and exclusion criteria before giving birth(9 because of preterm delivery, 238 selected epidural labor, 7 were given diazepam, 78 had gestational diabetes and 30 had gestational hypertension). 19(10.7%) were further excluded during delivery(1 was ransferred to cesarean delivery, 17 had postpartum hemorrhage, 1 neonatal Apgar score < 8 in 1 min). The remaining 159 women and newborns made up the original study cohort to be observed, but three newborns were excluded due to neonatal pathological jaundice affecting postnatal breastfeeding, two mothers did not return to the hospital for re-examination on time, and they were lost to follow-up. Therefore, 154 pairs of women and newborns constituted the final study cohort, Fig. 1 reports the complete recruiting flow diagram.

Those who remained were divided into four groups: (1) 39 pairs were given low dose of synOT (≤ 2.5 U), (2) 38 pairs were given a medium dose of synOT (> 2.5 U), (3) 38 pairs were given a high dose of synOT (> 7.5 U) and (4) 39 pairs were not given synOT (control group). Although grouped according to drug use after observation, results showed that mothers in the four cohorts were not significantly different in age (*p* = 0.359), gestational age (*p* = 0.974), BMI (*p* = 0.278), Habitation (*p* = 0.934), Educational background (*p* = 0.455), Monthly income (*p* = 0.709), parity (*p* = 0.664), Nipple condition (*p* = 0.258), Abortion history (*p* = 0.222), Total stage of labor (*p* = 0.071), Rupture of membranes time (*p* = 0.060), or Postpartum bleeding (*p* = 0.287). Newborns were not different regarding gender (*p* = 0.115), birth weight (*p* = 0.750) or one-minute Apgar (*p* = 0.532) (Table 2). By comparing the demographic data of mothers and newborns, the factors that may influence breastfeeding were balanced and comparable across the four groups and did not affect our analysis of the results.

**Table 2:** Mothers and infants baseline variables.

	Low-dose group (n = 39)	Medium-dose group (n = 38)	High-dose group (n = 38)	Control group (n = 39)	F/*χ*^2^	*P* value
**Mother**						
Age^a^	31.4 ± 4.1	30.6 ± 3.1	30.6 ± 3.3	29.9 ± 4.0	1.082	0.359
Gestational age^a^	39.2 ± 0.2	39.2 ± 0.8	39.2 ± 0.9	39.2 ± 0.9	0.073	0.974
BMI^a^	28.0 ± 3.1	28.2 ± 2.9	27.2 ± 1.8	27.4 ± 2.8	1.307	0.278
**Habitation**^b^						
Urban	37 (94.9)	35 (92.1)	36 (94.7)	36 (92.3)	0.430	0.934
Rural	2 (5.1)	3 (7.9)	2 (5.3)	3 (7.7)		
**Educational background**^b^						
College diploma below	5 (12.8)	3 (7.9)	3 (7.9)	3 (7.7)	8.812	0.455
College diploma	7 (17.9)	14 (36.8)	13 (34.2)	10 (25.7)		
Bachelor	20 (51.3)	19 (50.0)	20 (52.6)	23 (59.0)		
Master or higher	7 (17.9)	2 (5.3)	2 (5.3)	3 (7.7)		
**Monthly income (yuan)**^b^						
< 5000	2 (5.1)	2 (5.3)	1 (2.6)	1 (2.6)	8.925	0.709
5000~	1 (2.6)	3 (7.9)	2 (5.3)	2 (5.1)		
10,000~	6 (15.4)	4 (10.5)	8 (21.1)	11 (28.2)		
15,000~	19 (48.7)	18 (47.4)	13 (34.2)	18 (46.2)		
≥ 20,000	11 (28.2)	11 (28.9)	14 (36.8)	7 (17.9)		
**Primipara**^b^						
Yes	14 (35.9)	19 (50.0)	16 (42.1)	17 (43.6)	1.580	0.664
No	25 (64.1)	19 (50.0)	22 (57.9)	22 (56.4)		
**Nipple condition**^b^						
Normal	38 (97.4)	38 (100.0)	35 (92.1)	36 (92.3)	4.035	0.258
Retraction	1 (2.6)	0 (0.0)	3 (7.9)	3 (7.7)		
**Abortion history**^b^						
Yes	19 (48.7)	18 (47.4)	20 (52.6)	12 (30.8)	4.390	0.222
No	20 (51.3)	20 (52.6)	18 (47.4)	27 (69.2)		
Tolal stage of labor (min)^a^	315.4 ± 138.7	345.3 ± 142.7	367.4 ± 91.2	310.1 ± 114.2	2.427	0.071
Rupture of membranes (min)^a^	198 ± 7 ± 126.4	240.2 ± 122.6	214.5 ± 119.2	176.3 ± 82.9	2.570	0.060
Postpartum bleeding (ml)^a^	192.1 ± 51.2	193.2 ± 47.0	230.3 ± 147.4	215.6 ± 100.2	1.279	0.287
**Infant**						
Gender^b^						
Boy	19 (48.8)	16 (42.1)	26 (68.4)	19 (48.7)	3.932	0.115
Girl	20 (51.3)	22 (57.9)	12 (31.6)	20 (51.3)		
Birth weight (g)^a^	3367.2 ± 467.4	3444.5 ± 289.1	3469.0 ± 427.3	3410.0 ± 356.0	0.404	0.750
One-minute Apgar^a^	9.90 ± 0.3	9.97 ± 0.2	9.97 ± 0.2	9.95 ± 0.2	0.738	0.532

^a^Mean ± standard deviation.

^b^Frequency, percentage The total stage of labor refers to the entire process from the parturition (regular uterine contractions) to the delivery of the fetus and placenta.

### Birth cry, relaxation and awakening stage

There was no difference between the four groups in occurrences of movement and the duration times of birth cry, relaxation and awakening stages (Tables 3, 4).

**Table 3:** Occurrence time of newborn breastfeeding behaviors (min).

Stage	Behaviors	Low-dose group (n = 39)	Middle-dose group (n = 38)	High-dose group (n = 38)	Control group (n = 39)	*F*	*p* value
Birth cry	Crying (s)	3.8 ± 1.2	4.2 ± 1.3	3.7 ± 1.2	4.1 ± 1.5	1.250	0.294
Awakening	Opening eye	3.8 ± 1.1	3.8 ± 0.8	3.6 ± 0.8	3.7 ± 1.1	0.368	0.776
	Licking lips	8.2 ± 3.2	9.6 ± 2.5	8.9 ± 2.4	8.9 ± 3.6	1.333	0.270
Activity	Kicking legs	8.6 ± 2.4	9.8 ± 2.8	9.7 ± 2.1	9.7 ± 3.1	1.902	0.136
	Stretching arms	11.2 ± 1.3	12.1 ± 2.2	11.7 ± 2.1	10.8 ± 2.9	2.465	0.068
	Raising or turning head	15.2 ± 3.0	15.7 ± 3.0	15.9 ± 2.8	13.6 ± 3.2	4.655	0.004
	Touch nipples	21.4 ± 4.9	18.3 ± 6.3	21.0 ± 5.8	17.4 ± 7.1	2.410	0.072
	Eating hands	19.4 ± 3.1	20.2 ± 3.2	21.2 ± 3.3	17.6 ± 6.0	3.992	0.011
Clawing	Moving body	24.7 ± 4.1	24.4 ± 6.4	28.1 ± 5.4	22.8 ± 6.5	5.774	0.001
Familiari-zation	Locating areola	36.4 ± 6.7	38.1 ± 12.8	44.1 ± 12.0	33.5 ± 8.4	6.686	< 0.001
	Licking nipples	39.0 ± 7.5	42.5 ± 13.7	46.5 ± 12.6	36.7 ± 8.8	5.494	0.002
	Containing nipple	41.0 ± 7.6	43.7 ± 13.7	48.7 ± 13.3	37.8 ± 8.6	6.023	0.001
Sucking	Effective sucking	45.3 ± 6.8	47.3 ± 11.8	49.6 ± 13.5	39.5 ± 7.7	7.439	< 0.001
Sleeping	Falling asleep	84.8 ± 12.3	83.9 ± 9.0	88.4 ± 10.7	81.5 ± 11.5	1.537	0.212

The neonates in the relaxation and rest stages did not have any significant movements, which are not listed in the table.

**Table 4:** Duration time of newborn behavior stage (min).

Stage	Low-dose group (n = 39)	Middle-dose group (n = 38)	High-dose group (n = 38)	Control group (n = 39)	*F*	*p* value
Birth cry (s)	54.8 ± 7.1	54.3 ± 5.9	53.5 ± 6.5	51.3 ± 6.6	2.180	0.093
Relaxation	3.1 ± 0.6	2.9 ± 0.8	3.1 ± 0.9	3.0 ± 1.0	0.342	0.795
Awakening	4.6 ± 1.8	5.4 ± 1.8	5.5 ± 1.3	5.0 ± 2.0	0.441	0.070
Activity	15.7 ± 5.0	14.5 ± 6.8	18.1 ± 6.2	13.1 ± 6.3	4.655	0.004
Clawing	11.1 ± 4.3	11.5 ± 5.0	15.1 ± 5.2	11.2 ± 5.5	5.550	0.001
Familiarization	8.1 ± 3.9	9.5 ± 4.4	6.4 ± 2.7	5.8 ± 4.0	6.467	0.001
Sucking	34.6 ± 4.6	31.3 ± 5.0	30.8 ± 4.3	39.9 ± 4.9	28.988	< 0.001

The duration time of the sleep stage was not recorded due to the limitations of the observation time in the delivery room.

### Activity stage

**Figure 2:**
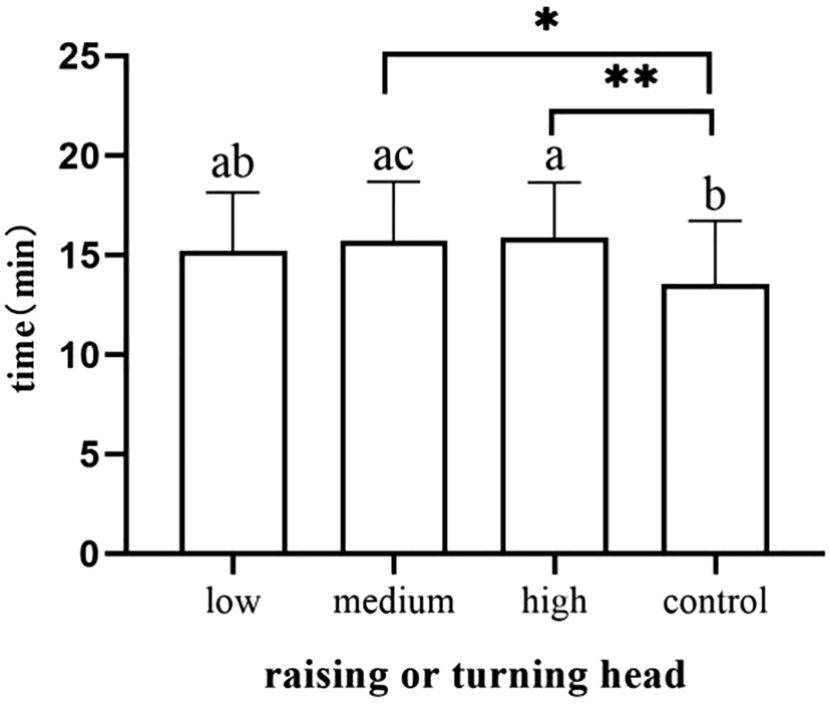
Occurrence time of raising or turning head.

**Figure 3:**
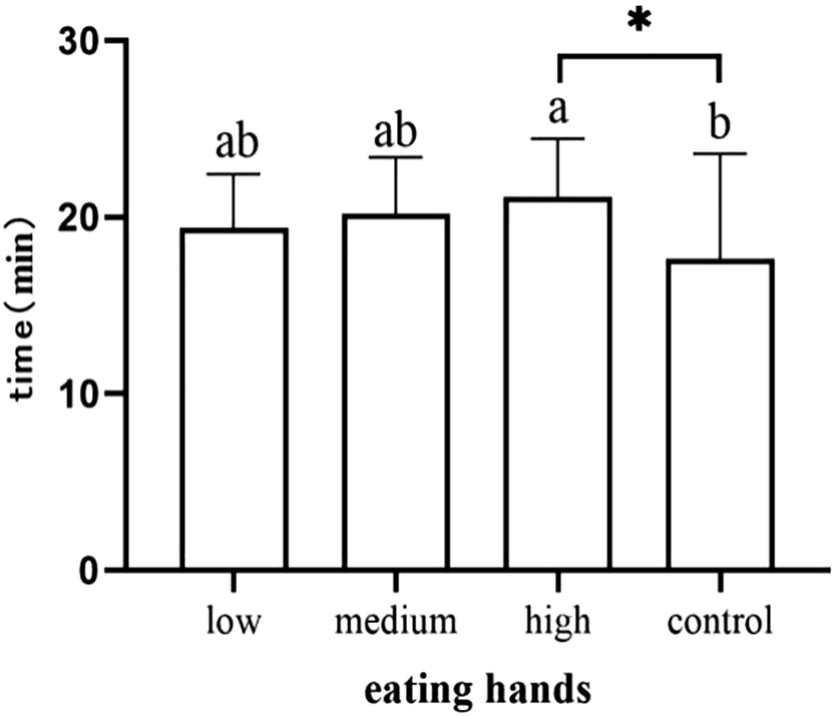
Occurrence time of eating hands.

**Figure 4:**
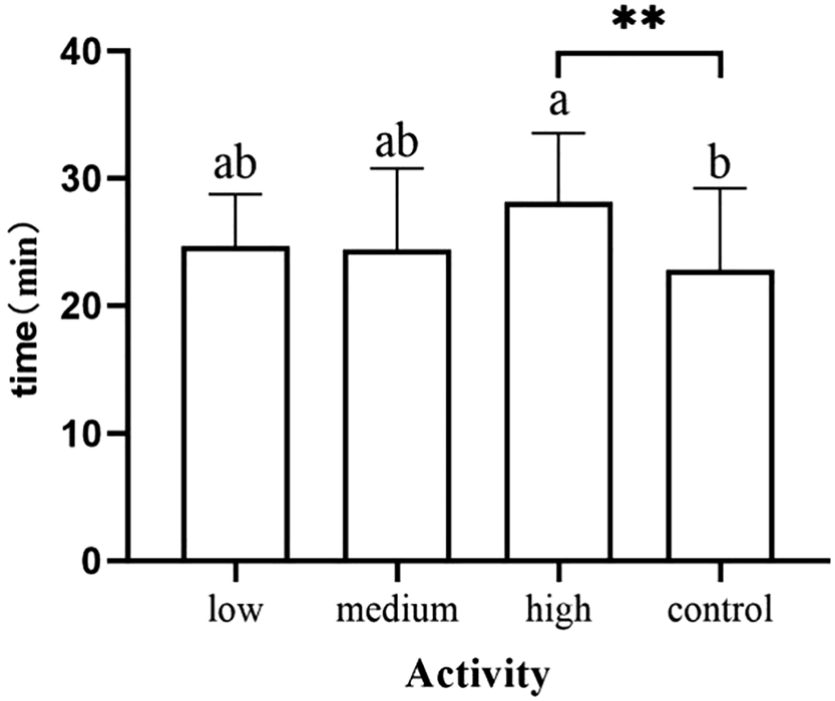
Duration of activity stage.

Newborns in the medium-dose group and high-dose group demonstrated head raising or head-turning significantly later than the control group (*p* = 0.013) (*p* = 0.006) (Fig. 2). Infants in the high dose group had significantly later time occurrences of eating their hands than the control group (*p* = 0.017) (Fig. 3). Infants in the high-dose group needed more time to pass through the activity stage than those in the control group (*p* = 0.003) (Fig. 4). We did not observe and record the resting stage because it was interspersed between the stages, and the newborns had no significant movement.

### Clawing stage

**Figure 5:**
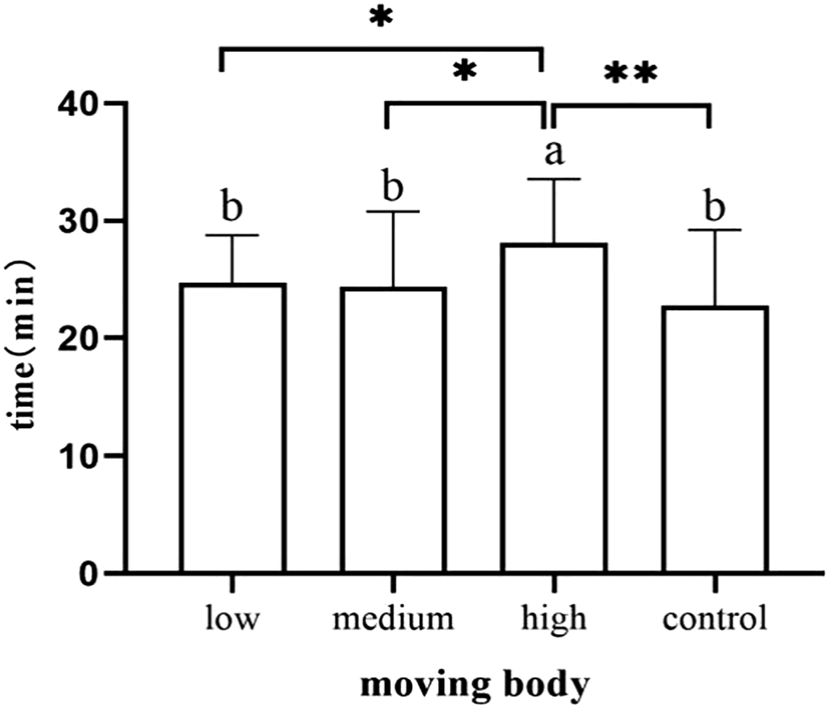
Occurrence time of moving body.

**Figure 6:**
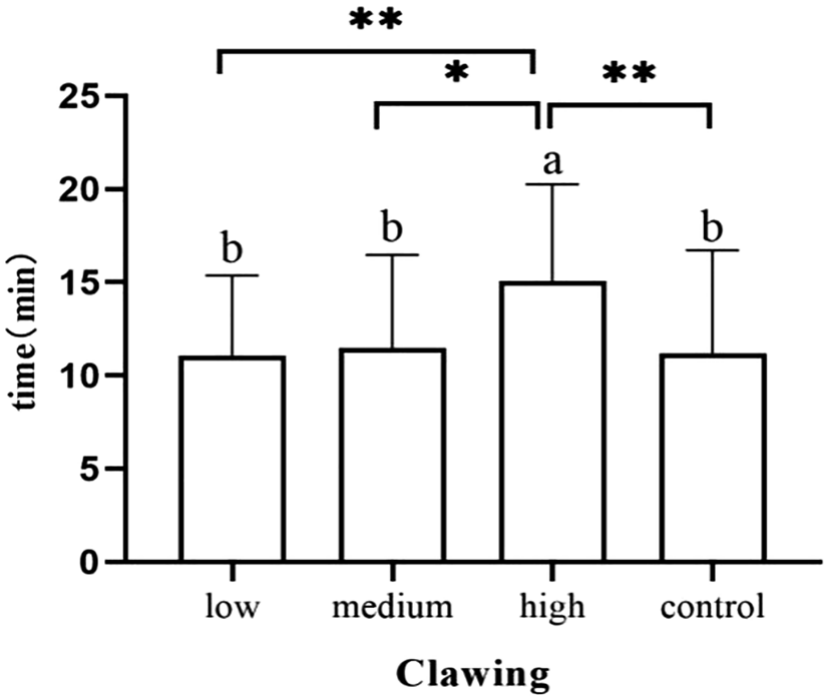
Duration of clawing stage.

Newborn body movement to the breast occurred later in the high-dose group than in the other groups, with groups ranked in the following way: newborns in the medium-dose group (*p* = 0.045), newborns in the low-dose group (*p* = 0.015) and newborns in the control group (*p* = 0.001) (Fig. 5). Additionally, it took longer for infants to reach the nipple in the high-dose group than in the medium-dose group (*p* = 0.013), newborns in the low-dose group (*p* = 0.004) and newborns in the control group (*p* = 0.006) (Fig. 6).

### Familiarization stage

**Figure 7:**
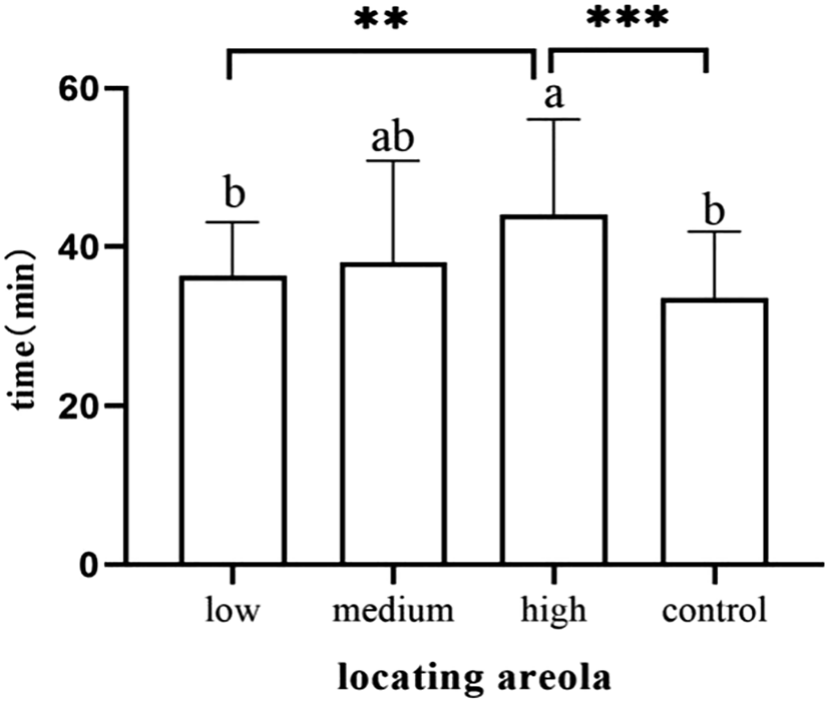
Occurrence time of locating areola.

**Figure 8:**
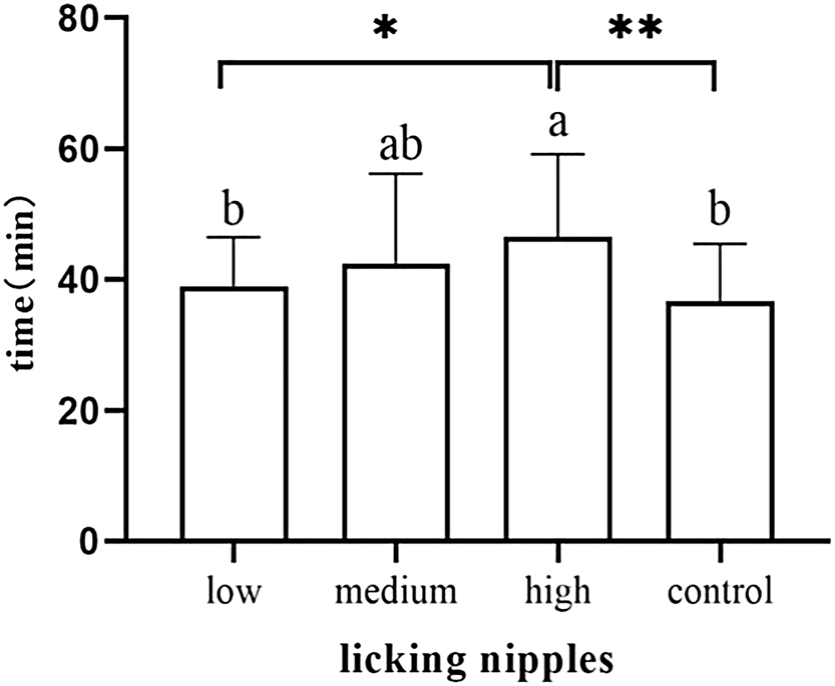
Occurrence time of licking nipples.

**Figure 9:**
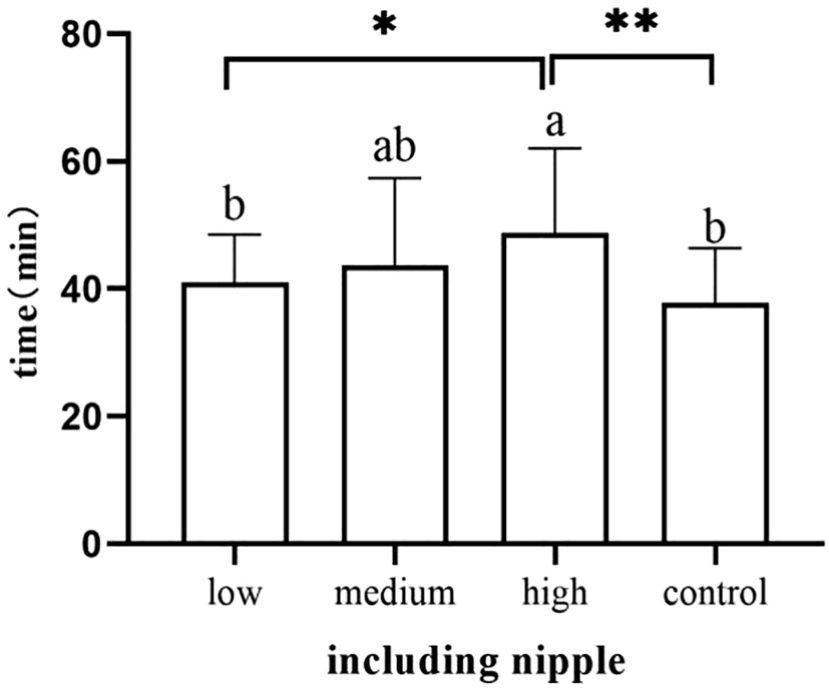
Occurrence time of including nipple.

**Figure 10:**
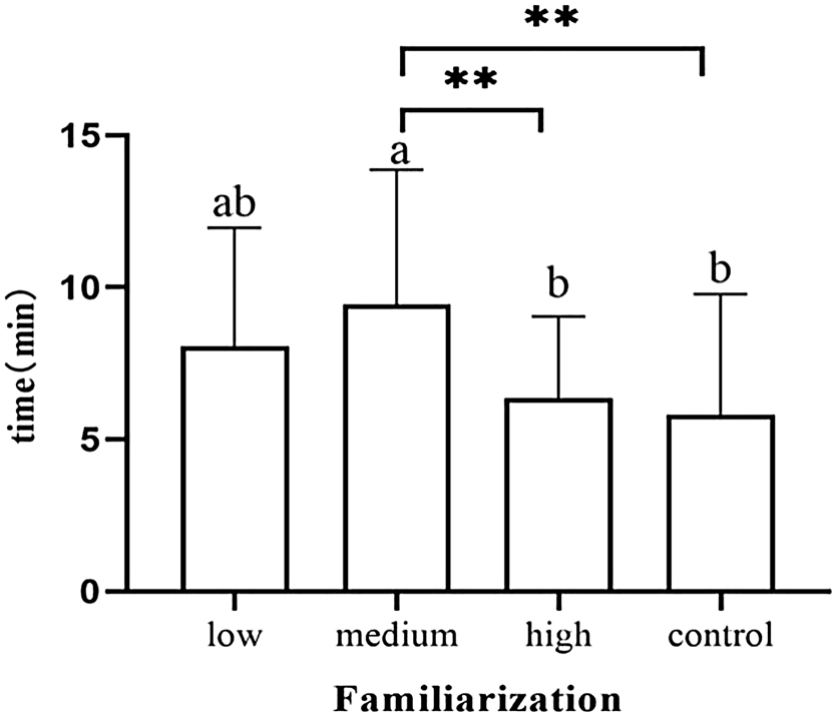
Duration of familiarization stage.

Neonates in the high-dose group showed significantly later areolar localization than the low-dose group (*p* = 0.006) and the control group (*p* < 0.001) (Fig. 7). Neonates in the high-dose group showed nipple licking significantly later than the low-dose group (*p* = 0.019) and the control group (*p* = 0.002) (Fig. 8).Newborns in the high-dose group showed significantly later nipple containing than the low-dose group (*p* = 0.022) and the control group (*p* = 0.001) (Fig. 9). Newborns in the medium-dose cohort needed a longer time in the familiarization stage than those in the high-dose group (*p* = 0.003) and control group (*p* = 0.002) (Fig. 10).

### Sucking stage

**Figure 11:**
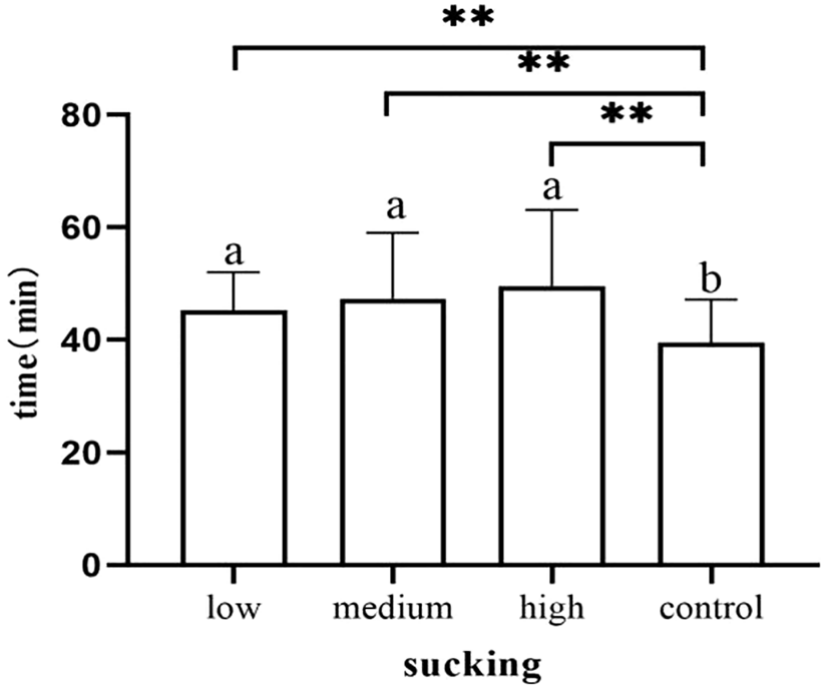
Occurrence time of sucking.

**Figure 12:**
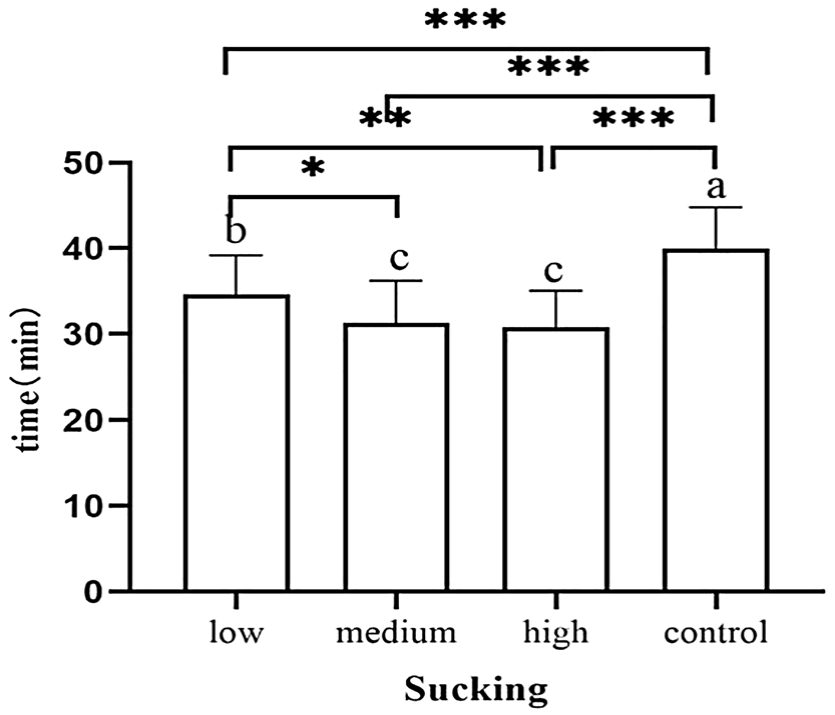
Duration of sucking stage.

The effective suckling time of neonates in the control group was earlier than that of neonates in the high-dose group (*p* = 0.002), medium-dose group (*p* = 0.007) and low-dose group (*p* = 0.005) (Fig. 11). The duration of neonatal sucking in the control cohort was longer than that in the high-dose, medium-dose and low-dose groups (*p* < 0.001). Newborns in the low-dose group also sucked longer than those in the medium-dose (*p* = 0.005) and high-dose group (*p* < 0.001) (Fig. 12).

### Sleeping stage

There was no difference between the four groups in occurrences of falling asleep (Table 3). We did not observe the duration of the sleeping stage due to the limitations of maternal and neonatal observation times in the delivery room.

### Comparison of neonatal breastfeeding

**Figure 13:**
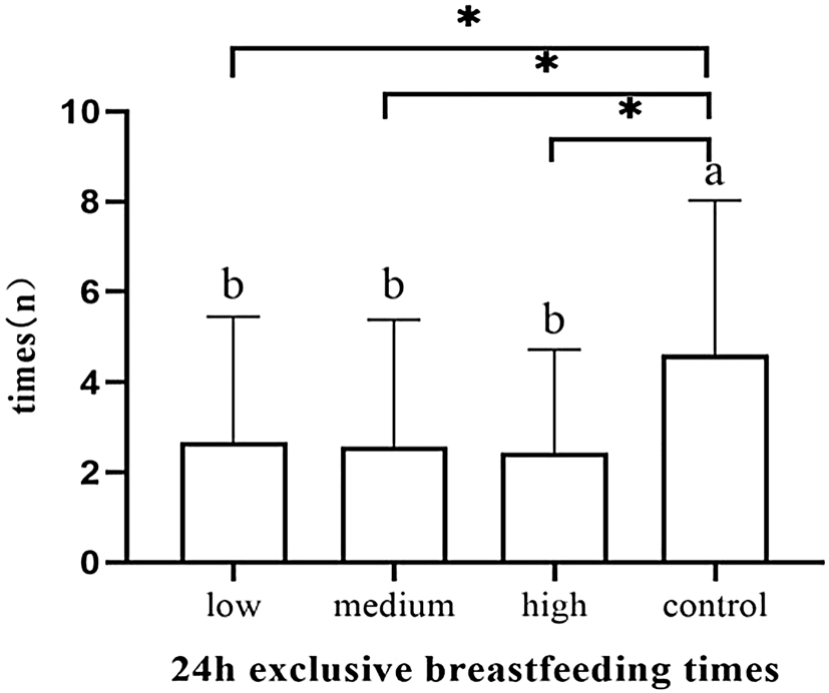
The number of exclusive breastfeeding sessions of 24 h.

**Figure 14:**
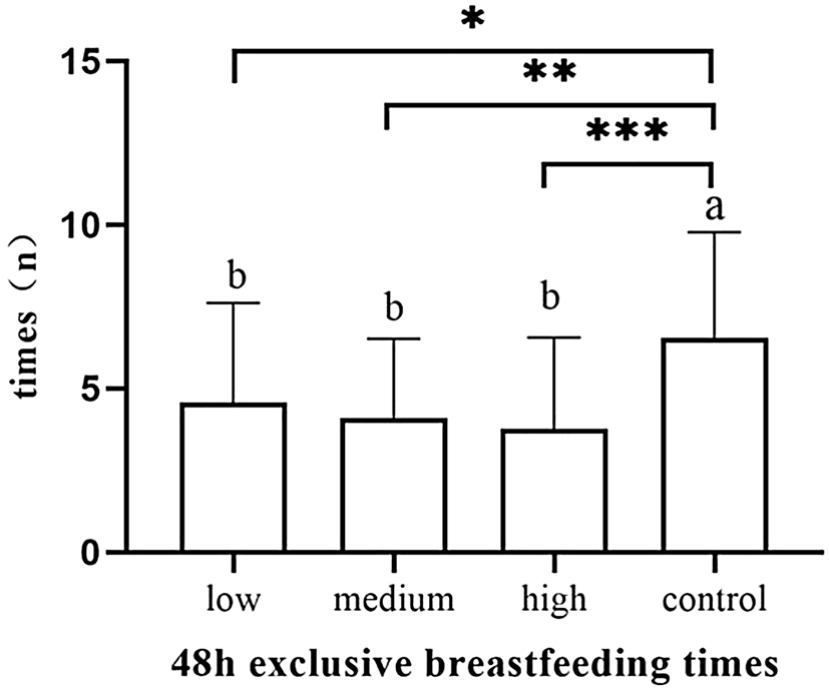
The number of exclusive breastfeeding sessions of 48 h.

**Figure 15:**
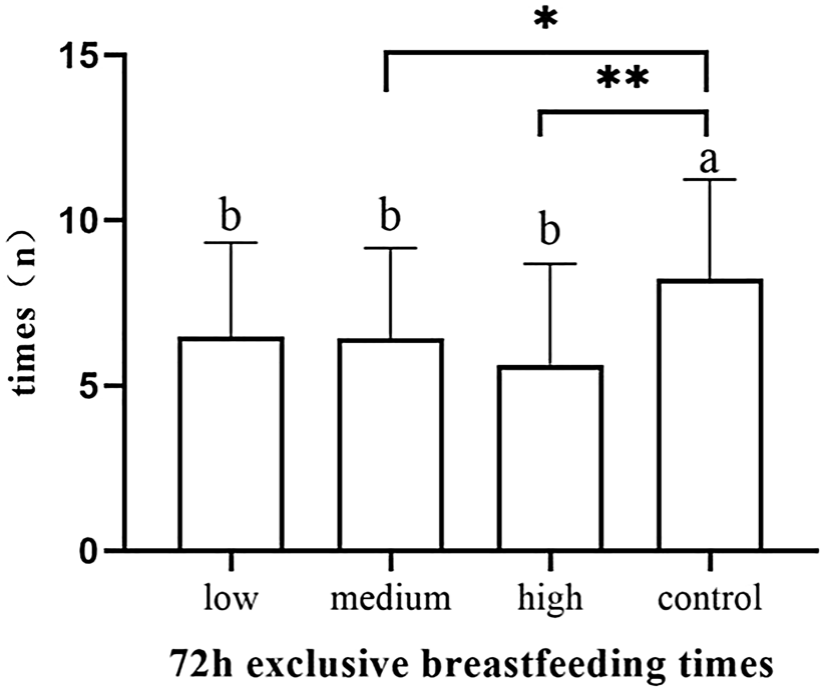
The number of exclusive breastfeeding sessions of 72 h.

The number of exclusive breastfeeding sessions of 24 h in the control group was significantly higher than the low-dose group (*p* = 0.042), the medium-dose group (*p* = 0.032) and the high-dose group (*p* = 0.01) (Fig. 13). The number of exclusive breastfeeding sessions of 48 h in the control group was significantly higher than the low-dose group (*p* = 0.018), the medium-dose group (*p* = 0.002) and the high-dose group (*p* < 0.001) (Fig. 14).The number of exclusive breastfeeding sessions of 72 h in the control group was significantly higher than the medium-dose group (*p* = 0.043) and high-dose group (*p* = 0.001) (Table 5). There was no statistical difference in the rate of exclusive breastfeeding at 3 months after birth among the four groups (Fig. 15) (Table 6).

**Table 5:** The number of exclusive breastfeeding sessions of 3 days (n).

Group	24 h (n)	48 h (n)	72 h (n)
Low-dose group (n = 39)	2.7 ± 2.8	4.6 ± 3.1	6.5 ± 2.8
Medium-dose group (n = 38)	2.6 ± 2.8	4.1 ± 2.4	6.4 ± 2.7
High-dose group (n = 38)	2.5 ± 2.3	3.8 ± 2.8	5.6 ± 3.0
Control group (n = 39)	4.6 ± 3.4	6.5 ± 3.2	8.2 ± 3.0
*F*	3.979	7.112	5.497
*p* value	0.011	< 0.001	0.001

**Table 6:** The rate of exclusive breastfeeding for 3 months after birth [n (%)].

Group	Exclusive breastfeeding	Partial feeding
Low-dose group (n = 39)	33 (84.6)	6 (15.4)
Medium-dose group (n = 38)	33 (86.8)	5 (13.2)
High-dose group (n = 38)	30 (78.9)	8 (21.1)
Control group (n = 39)	34 (87.2)	5 (12.8)
*x*^*2*^	1.261	
*p* value	0.738	

## Discussion

Although previous studies have focused on the potential effects of synOT on newborn behavior^12,14,15,20^ and breastfeeding^11,21^, this study further analyzed the effects of different doses of synOT on newborn breast seeking movement and behavior stages. Our results suggest that synOT may affect neonatal instinctive behaviors and breastfeeding after birth, and the associations can vary with different doses.

Previous studies have shown that synOT can reach the fetal brain through both the placental barrier and the fetal brain-blood barrier, thus potentially interfering with the development of the fetal endogenous oxytocin system^22,23^. Although synOT has a short half-life in circulation, as well as the fact that only a small amount crosses the blood–brain barrier, prolonged synOT infusion inhibits the release of endogenous oxytocin's physiological impulses, which can possibly lead to changes in the neuroendocrine environment of the neonatal brain as it continues to develop, thus interfering with the development of the fetal endogenous oxytocin system and the function of the oxytocin receptor. Overall, this can lead to changes in neonatal behavior^24^. Endogenous oxytocin is a paraventricular neuroendocrine peptide composed of 9 amino acids and synthesized by the hypothalamus. It then travels to the pituitary gland, where it is stored; additionally,endogenous oxytocin has been shown to enhance the activation of brain regions associated with connectivity and empathy, as well as functional connections between these regions. It also plays an important role in the development of parental behavior^8,25,26^. SynOT enters the fetal brain, which saturates brain receptors, interferes with endogenous oxytocin secretion and the availability of endogenous oxytocin receptors and possibly alters the newborn's behavior^21^. Newborns in the control group exhibited the earliest time in raising and turning their heads, as well as in eating their hands, moving their bodies, contacting the areola with lips, licking nipples, containing nipples, sucking and falling asleep. The shortest time to progress through the stages of activity, crawling and familiarization, as well as sucking, lasted the longest.

The occurrence of head raising or head turning in the medium-dose group and high-dose group were significantly later than those in the control group. Additionally, newborns in the high-dose group had higher latencies of hand eating than those in the control group, and lower doses had no significant effect on these movements. Rooting activity (head turning to one side with an open mouth), hand swiping at the mouth and hand-to-mouth contact are indicators of prefeeding cues, and fewer cues were observed in infants exposed to synOT^14^. Our results suggest that medium and high doses of synOT can delay neonatal prefeeding cues. The presence of prefeeding cues in newborns was associated with the success of breastfeeding within the first hour after birth, also reflects the emerging neonatal neurobehavioral ability, can more easily from the crying or unstable state transition to more calm behavior, it also suggests that neonatal neurobehavioral development may be sensitive to the oxytocin^27^. The activity stage duration was substantially longer in the high-dose cohort than in the control group, which may be because the delay in the occurrence of movement prolonged the behavior stage.

Newborns in the high-dose cohort had significantly later body movements than other three groups and took substantially longer to crawl to their mothers' breasts than other three groups. Kajsa Brimdyr et al.^20^ observed that newborns who were exposed to synOT needed the longest time for the crawling stage. The study found that newborns who started crawling earlier were more likely to find their mothers' nipples and areola in a shorter period^17^, high-dose synOT could affect neonatal breast-searching activities, which leads to a later arrival at the mother's breast.

The occurrence times of the lips locating the areola, licking the nipple and insertion of the nipple were later in the high dose cohort; in addition, newborns in the medium-dose cohort took more time in the familiarization stage. This is different from previous studies, and we think that prolonged familiarization may be related to decreased duration of rest. SynOT exposure has been found to reduce rest time in the first hour of neonates^20^. Areas of the hippocampus and cortex are more active at times of rest^28^, and the hippocampus, with its clusters of OTRs, is the part of the brain associated with solidifying memories and social behavior and bonding^29,30^. Less rest time is not conducive to consolidating memories and facilitating self-attachment of breasts in newborns^31,32^ and it can also affect the start of breastfeeding. Low doses of synOT favor memory, social learning and maternal behavior. However, high doses of synOT may interfere with social memory^33^. We did not record the rest times of infants, and the association between rest time and synOT dose needs further study. At this stage, neonatal licking and massage on the nipple or areola can promote the nipple erection and easy attachment, and also promote the release of maternal synOT and lactation. However, in the high-dose group, the late occurrence of locating, licking the nipple and lactation may affect the time of first successful sucking and early lactation of the mother.

In comparison with the control group, newborns in the other three groups started sucking at significantly later time points, and the high dose group was the last group to initiate sucking. The duration of the sucking stage of newborns in the control cohort was significantly longer than that in the other three groups. Moreover, there was a significantly longer survival time in the low-dose group than in the medium- and high-dose groups. Olza Fernández et al.^12^ observed that exogenous oxytocin interferes with sucking and breastfeeding duration and that the synOT dose was negatively correlated with neonatal sucking. This is consistent with our findings. Additionally, we observed that low doses of synOT may affect the sucking of the newborn, the effect becomes more pronounced with increasing doses, and the duration of the first sucking decreases as the dose increases.

Infant breastfeeding behavior scores, such as rooting, latch-on, sucking, swallowing and activity states, were correlated with neurobehavioral function scores^34^. The adverse effects of oxytocin on neonatal breast searching and sucking should compel us to consider the impact of synOT on neonatal neural behavior. The current study found that boys exposed to synOT for the longest period and with the highest cumulative dose had a significantly higher chance of being diagnosed with autism^35^. Further investigation is still needed to fully elucidate the short-term and long-term effects of synOT on neonatal neurobehavior.

The first two hours after birth have been described as the critical period for stable breastfeeding and its continuity, the sensitive period for establishing effective breastfeeding^36^. Early breastfeeding promotes the production of breast milk, and its practice determines the successful establishment and duration of breastfeeding^37^. Our results suggest that low, medium and high doses of synOT may lead to a significant reduction in the number of exclusive breastfeeding sessions within 24 h after birth. The administration of synOT results in desensitization of oxytocin receptors, which inhibits the maternal endogenous oxytocin release and endogenous circulating oxytocin production through a negative feedback mechanism^29^. Maternal endogenous oxytocin stimulates the contraction of mammary myoepithelial cells to produce lactation, and the decrease of endogenous oxytocin will affect the time of lactation. Jonas and colleagues demonstrated that the mothers who had received the highest doses of synOT released the lowest amount of their endogenous oxytocin in the second day postpartum^9^. The number of exclusive breastfeeding sessions of 48 h in the low, medium and high dose group were significantly less than those in the control group , with the increase of dose, the effect was more significant. On the third day after delivery, the number of exclusive breastfeeding sessions in the medium, high dose group was less. We speculated that the time of lactation in the low-dose group was earlier than the medium and high dose group, so the session of exclusive breastfeeding was increased.

The study showed that the use of synOT increased the risk of milk feeding later by 1.451 times^21^. Olza Fernandez I found a negative association between synOT dose and exclusive breastfeeding for 3 months after birth^12^. However, some studies suggest that the synOT is not associated with the exclusive breastfeeding of newborns at 1 month, 3 months and 6 months after discharge^38^, Gomes also showed that fewer babies were exclusively breastfed during the first 3 months of life among mothers who used synOT, but there was no correlation between synOT and rates of exclusive breastfeeding at the third month^39^. Our results also showed that the high-dose group had lower rates of exclusive breastfeeding at 3 months than the control group, but there was no difference. We believe that the first reason is the encouragement and advocacy of hospitals and society for breastfeeding. Most puerpera and their families are relatively supportive of exclusive breastfeeding. Second, due to the small sample size, the finding of no difference in rates of exclusive breastfeeding at 3 months may be underrepresentative, further analysis should be made based on expanding the sample size.

This study analyzed the effects of different doses of synOT on neonatal instinctive behavior, suggesting that low doses of synOT may have adverse effects on the early sucking of neonates, and with the increase of the dose of synOT, the associations with neonatal breast search activity and breast attachment will be more significant. The use of low, medium and high doses of synOT during delivery reduced the number of exclusive breastfeeding sessions within 3 days after birth and may adversely affect the continuity of breastfeeding.

## Limitations

First, this study did not collect information about neonatal neurological development, and we obtained the times of newborn movement through observation, which is subjective. We were only able to train the researchers in relevant knowledge before the study, and two researchers determined the times in the study to ensure objectivity. Second, before the study began, we screened for factors that might affect neonatal behavior through reviewing the literature and clinical practice, incorporating these factors into exclusion criteria and baseline data, and excluding or equalizing confounding factors so that the four groups were comparable. However, there may still be other confounding factors in the study affecting the outcome of breastfeeding, and we will conduct comprehensive analysis in further study. Third, the length of exposure to the same concentration of synOT may also be an important indicator, and analysis will be conducted in a subsequent study.

## Conclusions

Intrapartum application of different doses of synOT may affect the full expression of neonatal instinctive behavior within 2 h after birth, and adverse to the initiation and continuation of breastfeeding. This study provides a reference for the optimal application of synOT in clinical practice and proves the necessity of studying the effects of synOT on neonatal neurobehavioral development. Further studies should focus on the associations of synOT and its dose on neonatal nervous system and psychobehavioral development.

## Data Availability

All data generated or analysed during this study are included in this published article.

## References

[1] du Vigneaud V (1953). The synthesis of an octapeptide amide with the hormonal activity of oxytocin. J. Am. Chem. Soc..

[2] Simpson KR, Knox GE (2009). Oxytocin as a high-alert medication: Implications for perinatal patient safety. MCN Am. J. Matern. Child Nurs..

[3] Pitocin (Oxytocin Injection, USP) Synthetic label. Cited (2017).

[4] Page K, McCool WF, Guidera M (2017). Examination of the pharmacology of oxytocin and clinical guidelines for use in labor. J. Midwifery Womens Health..

[5] Wiberg-Itzel E, Pembe AB, Wray S (2014). Level of lactate in amniotic fluid and its relation to the use of oxytocin and adverse neonatal outcome. Acta Obstet. Gynecol. Scand..

[6] Bakker PC, Kurver PH, Kuik DJ, Van Geijn HP (2007). Elevated uterine activity increases the risk of fetal acidosis at birth. Am. J. Obstet. Gynecol..

[7] Bell AF, Erickson EN, Carter CS (2014). Beyond labor: the role of natural and synthetic oxytocin in the transition to motherhood. J. Midwifery Womens Health..

[8] Carter CS (2013). Developmental consequences of oxytocin. Physiol. Behav..

[9] Jonas W (2009). Effects of intrapartum oxytocin administration and epidural analgesia on the concentration of plasma oxytocin and prolactin, in response to suckling during the second day postpartum. Breastfeed Med..

[10] Gimpl G, Fahrenholz F (2001). The oxytocin receptor system: Structure, function, and regulation. Physiol. Rev..

[11] Odent MR (2013). Synthetic oxytocin and breastfeeding: Reasons for testing an hypothesis. Med. Hypotheses..

[12] Olza Fernández I (2012). Newborn feeding behaviour depressed by intrapartum oxytocin: A pilot study. Acta Paediatr..

[13] Bales KL (2007). Oxytocin has dose-dependent developmental effects on pair-bonding and alloparental care in female prairie voles. Horm Behav..

[14] Bell AF, White-Traut R, Rankin K (2013). Fetal exposure to synthetic oxytocin and the relationship with prefeeding cues within one hour postbirth. Early Hum. Dev..

[15] Marín Gabriel MA, Olza Fernández I, Malalana Martínez AM (2015). Intrapartum synthetic oxytocin reduce the expression of primitive reflexes associated with breastfeeding. Breastfeed Med..

[16] Dani C (2015). Behavior of the newborn during skin-to-skin. J. Hum. Lact..

[17] Widström AM (2011). Newborn behaviour to locate the breast when skin-to-skin: A possible method for enabling early self-regulation. Acta Paediatr..

[18] Bystrova K (2009). Early contact versus separation: Effects on mother-infant interaction one year later. Birth.

[19] Obstetrics Group. Branch of Obstetrics and Gynecology. Chinese Medical Association. Guidelines for cervical maturation and labor induction in the third trimester of pregnancy. *Chin. J. Obstet. Gynec.* **49**, 881–885 (2014).

[20] Brimdyr K, Cadwell K, Widström AM, Svensson K, Phillips R (2019). The effect of labor medications on normal newborn behavior in the first hour after birth: A prospective cohort study. Early Hum. Dev..

[21] García-Fortea P (2014). Oxytocin administered during labor and breast-feeding: A retrospective cohort study. Matern. Fetal Neonatal. Med..

[22] Saunders NR, Habgood MD, Dziegielewska KM (1999). Barrier mechanisms in the brain, II. Immature brain. Clin. Exp. Pharmacol. Physiol..

[23] Patient C, Davison JM, Charlton L, Baylis PH, Thornton S (1999). The effect of labour and maternal oxytocin infusion on fetal plasma oxytocin concentration. Br. J. Obstet. Gynaecol..

[24] Rydén G, Sjöholm I (1969). Half-life of oxytoxin in blood of pregnant and non-pregnant woman. Acta Obstet. Gynecol. Scand..

[25] Gordon I, Martin C, Feldman R, Leckman JF (2011). Oxytocin and social motivation. Dev. Cogn. Neurosci..

[26] Scatliffe N, Casavant S, Vittner D, Cong X (2019). Oxytocin and early parent-infant interactions: A systematic review. Int. J. Nurs. Sci..

[27] Ransjö-Arvidson AB (2001). Maternal analgesia during labor disturbs newborn behavior: Effects on breastfeeding, temperature, and crying. Birth.

[28] Tambini A, Ketz N, Davachi L (2010). Enhanced brain correlations during rest are related to memory for recent experiences. Neuron.

[29] Cadwell K, Brimdyr K (2017). Intrapartum administration of synthetic oxytocin and downstream effects on breastfeeding: Elucidating physiologic pathways. Ann. Nurs. Res..

[30] Feldman R (2012). Oxytocin and social affiliation in humans. Horm Behav..

[31] Craig M (2016). Comparable rest-related promotion of spatial memory consolidation in younger and older adults. Neurobiol. Aging..

[32] Edmond KM (2016). Delayed breastfeeding initiation increases risk of neonatal mortality. Pediatrics.

[33] EKlenerova V (2009). Oxytocin and carbetocin effects on spontaneous behavior of male rats. Neuro Endocrinol. Lett..

[34] Radzyminski S (2005). Neurobehavioral functioning and breastfeeding behavior in the newborn. J. Obstet. Gynecol. Neonatal Nurs..

[35] Soltys SM, Scherbel JR, Kurian JR (2020). An association of intrapartum synthetic oxytocin dosing and the odds of developing autism. Autism.

[36] Sharma A (2016). Efficacy of early skin-to-skin contact on the rate of exclusive breastfeeding in term neonates: A randomized controlled trial. Afr. Health Sci..

[37] Takahashi K, Ganchimeg T, Ota E (2017). Prevalence of early initiation of breastfeeding and determinants of delayed initiation of breastfeeding: Secondary analysis of the WHO Global Survey. Sci. Rep..

[38] Fernández-Cañadas Morillo A (2017). The relationship of the administration of intrapartum synthetic oxytocin and breastfeeding initiation and duration rates. Breastfeed Med..

[39] Gomes M (2018). Intrapartum synthetic oxytocin and breastfeeding: A retrospective cohort study. J. Obstet. Gynaecol..

